# Exploring the Neuroprotective Role of Selenium: Implications and Perspectives for Central Nervous System Disorders

**DOI:** 10.1002/EXP.20240415

**Published:** 2025-04-01

**Authors:** Guanning Huang, Ying Liu, Xiadong Zhu, Lizhen He, Tianfeng Chen

**Affiliations:** ^1^ Department of Neurology and Stroke Center of The First Affiliated Hospital College of Chemistry and Materials Science Key Laboratory for Regenerative Medicine of Ministry of Education Jinan University Guangzhou China

**Keywords:** nervous system disorders, neuroprotection, selenium, selenoproteins

## Abstract

Selenium (Se) is a crucial element in selenoproteins, key biomolecules for physiological function in vivo. As a selenium‐rich organ, the central nervous system can express all 25 kinds of selenoproteins, which protect neurons by reducing oxidative stress and inflammatory response. However, decreased Se levels are prevalent in a variety of neurological disorders, which is not conducive to the treatment and prognosis of patients. Thus, the biological study of Se has emerged as a focal point in investigating the pivotal role of trace elements in neuroprotection. This paper presents a comprehensive review of the pathogenic mechanism of neurological diseases, the protective mechanism of Se, and the neurological protective function of selenoproteins. Additionally, the application of Se as a neuroprotective agent in neurological disorder therapy, including ischemic stroke, Alzheimer's, Parkinson's, and other neurological diseases, is summarized. The present review aims to offer novel insights and methodologies for the prevention and treatment of neurological disorders with trace Se, providing a scientific basis for the development of innovative Se‐based neuroprotectants to promote their clinical application against neurological diseases.

## Graphical abstract

Selenium (Se) is a crucial element in selenoproteins, key biomolecules for physiological function in vivo. Central nervous system can express all 25 kinds of selenoproteins, which protect neurons by reducing oxidative stress and inflammatory response. Neuroprotection is being investigated through the biological study of Se. In this review, novel insights are presented for the prevention and treatment of neurological disorders with trace Se, providing scientific evidence for the development of innovative Se‐based neuroprotectants.

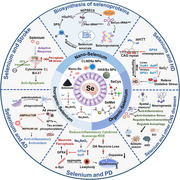

## 1 Introduction

Selenium (Se) is an essential trace element found in the human body that plays a physiological role by inserting selenoproteins through the 21^st^ necessary amino acid, selenocysteine (Sec) [1]. In the synthesis of selenoproteins, Sec is encoded by the codon UGA, which is transferred to the synthetic selenoprotein polypeptide chain by selenocysteine transporter RNA (tRNA) under specific translation conditions [2, 3, 4]. In 1957, Schwarz et al. found that a lack of Se in food could cause liver necrosis in rats, first revealing the protective effects of Se on the liver and its role in animal nutrition. Keshan disease, found in northeast China, is a myocardial disease caused by patients susceptible to the Coxsackie virus due to reduce glutathione peroxidase1 expression induced by dietary Se deficiency [5]. The World Health Organization confirmed Se as an essential trace element for humans, recommending a daily intake of 40 µg to effectively prevent the incidence of a variety of diseases [6]. Subsequent studies have confirmed the importance of selenoproteins in maintaining oxidation/reduction balance, promoting neuronal development, and regulating immune function and other physiological processes [7, 8, 9, 10, 11].

**FIGURE 1:**
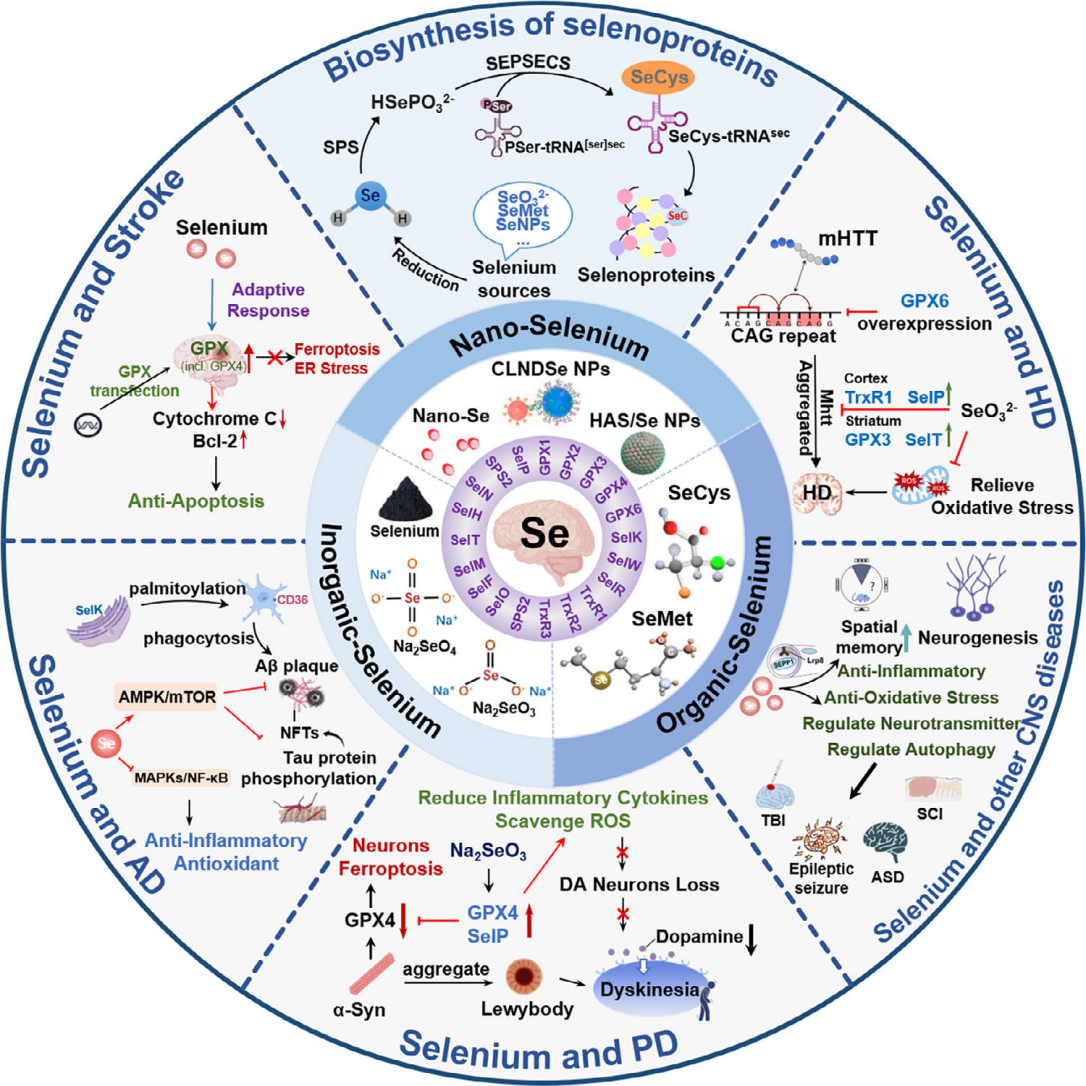
Application and mechanism of Se in neurological diseases.

Brain is the nervous system's central organ and is highly riched with Se. The dietary deficiency of Se can lead to reduced Se content and lower expression levels of selenoproteins in many organs, but the brain tissue can still maintain high Se content and a stable level of selenoproteins [12]. Moreover, the Se content in the brain is the first to return to normal levels after Se supplementation. This reveals that the brain initiatively maintains Se content for special regulatory mechanisms. Due to their high oxygen consumption, easy oxidation of polyunsaturated fatty acids, and relative lack of antioxidant enzymes, cerebral nerve cells in the brain are susceptible to the oxidative damage of reactive oxygen species (ROS) [13]. More than half of selenoproteins in the body have an antioxidant function and redox regulation ability, with their reduction of causing neuronal damage, cognitive impairment, depression, and anxiety [14, 15]. Based on the strong correlation between the occurrence of nervous system diseases and the expression of selenoproteins, this review summarizes the role of Se and provides a new direction for the study of neuroprotection (Figure 1).

## 2 Biological Functions of Selenium in Neurological Disease

It is estimated that 25 selenoproteins are present in the human body at present, including glutathione peroxidase1 (GPX1), glutathione peroxidase2 (GPX2), glutathione peroxidase3 (GPX3), glutathione peroxidase4 (GPX4), glutathione peroxidase6 (GPX6), thioredoxin reductase1 (TrxR1), thioredoxin reductase2 (TrxR2), thioredoxin reductase3 (TrxR3), iodothyronine deiodinases1 (DIO1), iodothyronine deiodinases2 (DIO2), iodothyronine deiodinases3 (DIO3), methionine‐Rsulfoxide reductase B1 (SelR), selenophosphate synthetase 2 (SPS2), selenoproteins F (SelF), selenoproteins H (SelH), selenoproteins I (SelI), selenoproteins K (SelK), selenoproteins N (SelN), selenoproteins M (SelM), selenoproteins O (SelO), selenoprotein P (SelP), selenoproteins V (SelV), selenoproteins S (SelS), selenoproteins T (SelT), and selenoproteins W (SelW) [16, 17]. Different selenoproteins types have different biological functions (Table 1). For example, GPXs such as glutathione peroxidase 4 (GPX4) can remove toxic metabolites such as hydrogen peroxide, lipid peroxides, and organic peroxides from the human cell membrane and cytoplasm, demonstrating the potential antioxidant function of Se [18, 19, 20]. TrxR reduces thioredoxin (Trx) via nicotinamide adenine dinucleotide phosphate to initiate oxidation/reduction reactions [21]. In addition, SelT has a similar enzymatic mechanism to TrxR [22]. SelP‐enriched plasma is not only responsible for the storage and transport of Se in vivo but also functions as an antioxidant role in protecting vascular endothelial cells [23]. Furthermore, SelS localized in the endoplasmic reticulum (ER) not only reduces ER stress but also relieves inflammation [24]. Previous studies have confirmed the importance of these antioxidant selenoproteins in protecting neurons and astrocytes from oxidative damage [25, 26, 27, 28]. Neurological diseases, including strokes, Parkinson's disease (PD), Alzheimer's disease (AD), Huntington's disease (HD), have been correlated with increased oxidative stress [29, 30]. Hence, selenoproteins deficiency or decreased selenoprotein activity are both closely linked to the biological function of Se in neurological diseases [31, 32]. Herein, the relationship between Se and the occurrence and development of nervous system diseases is summarized, providing a reference for the development of new strategies to utilize the biological activities of Se to treat neurological diseases.

**TABLE 1:** Role of selenoproteins in neurological diseases.

Selenoprotein	Biological functions	Involved diseases	Influencing factors	Reference
GPX	Enhance antioxidant capacity; Alleviate endoplasmic reticulum stress; Inhibit ferroptosis; Predict the prognosis of brain tumors.	AD, PD, Stroke, Glioma	GPX1 knockout Database analysis	[33, 34, 35, 36]
TrxR	Maintain REDOX balance; Regulate NO signaling.	PD, Epilepsy	Z‐ligustilide	[37, 38, 39]
SelP	Transport Se; Enhance antioxidant capacity.	AD, PD, Stroke	Ischemia/Reperfusion SelP knockout	[27, 40, 41]
SPS2	Se donor; Catalyze synthesis of monoselenophosphate (MSP);	−	SPS2 knockout	[42]
SelK	Maintain REDOX balance; Enhance immune function; Alleviate ER stress; Inhibit inflammatory; Maintain Ca^2+^ homeostasis;	AD, HD, PD	SelK knockout Na_2_SeO_3_	[9, 43, 44]
SelM	Maintain REDOX balance; Maintain Ca^2+^ homeostasis.	AD, HD	Na_2_SeO_3_ SelM knockout	[45, 46, 47]
SelR	Reduce methionine sulfoxide levels	AD, PD	−	[48]
SelS	Maintain REDOX balance; Alleviate ER stress; Involve in the degradation of ER related proteins; Inhibit inflammatory.	AD	Silencing SelS Na_2_SeO_4_	[49, 50, 51]
SelT	Facilitate *n*‐glycosylation; Alleviate ER stress.	PD	SelT knockout	[52]
SelW	Maintain REDOX balance; Protect embryonic neuron cells.	AD	SelP knockout	[53]

### 2.1 Application of Selenium in Ischemic Stroke

Patients experiencing stroke can only benefit from thrombolysis and vascular recirculation within 4.5 h of onset [54, 55]. In an emergency, prompt hospital admission for conventional intravenous thrombolysis is essential to recovery [54, 56]. Ischemic stroke is a common type of stroke that has become the second most lethal globally [57, 58]. According to the characteristics of stroke pathogenesis, the clinical treatment of ischemic stroke primarily focuses on the use of thrombolytic medicines to increase blood flow and neuroprotective therapy [59, 60, 61]. For example, Edaravone is a small molecule antioxidative drug used to repair brain oxidative injury, but its short half‐life and low bioavailability greatly limit its efficacy and clinical application [62, 63, 64, 65, 66]. Following blood flow obstruction in the stroke‐affected cerebral area, the oxygen and glucose requirements are not satisfied in vivo [58]. This causes a complex chain of cellular events, including oxidative stress, neuroinflammation, neuroexcitatory toxicity, and apoptosis, leading to neuronal injury and death [67]. For example, *N*‐methyl‐d‐aspartate receptor (NMDAR)‐mediated calcium influx has been shown to activate nitric oxide (NO) synthase [68]. In addition to elevated NO levels, a stroke can generate toxic chemicals, such as ROS, by stimulating xanthine oxidase and altering the electron transport chain in the mitochondria [69, 70].

Selenoproteins and other Se compounds have been considered in the treatment of ischemia/reperfusion injury owing to the antioxidant activity of Se [71]. Sodium selenite inhibits the reversal of mitochondrial membrane potential and ROS generation, notably reducing glutamate toxicity and hypoxia‐induced cell death in mouse hippocampus cells (HT22 cell line). According to Peroal et al., the risks of ischemic stroke were increased by mutations of the SelS gene, which upregulated the expression of inflammatory markers [72]. This was determined by examining changes in the expression level of SelS after cerebral ischemia‐reperfusion. Additionally, Joseph Loscalzo et al. reported that the deletion of GPX1 increased the amount of neuronal injury in MCAO mice models, which was represented by an increase in the number of TdT‐mediated dUTP nick end labeling positive cells in the brain, the size of the cerebral infarction, and the level of lipid hydrogen peroxide [73]. Ebselen is an organic Se molecule drug with GPX enzyme‐like activity that has shown excellent potential in clinical trials for the treatment of multiple systemic diseases [74, 75, 76]. Huang et al. observed that GPX1^−/−^ MCAO mice treated with ebselen showed distinct recovery in cerebral blood flow, blood–brain barrier permeability, and the area of cerebral infarction [77].

**FIGURE 2:**
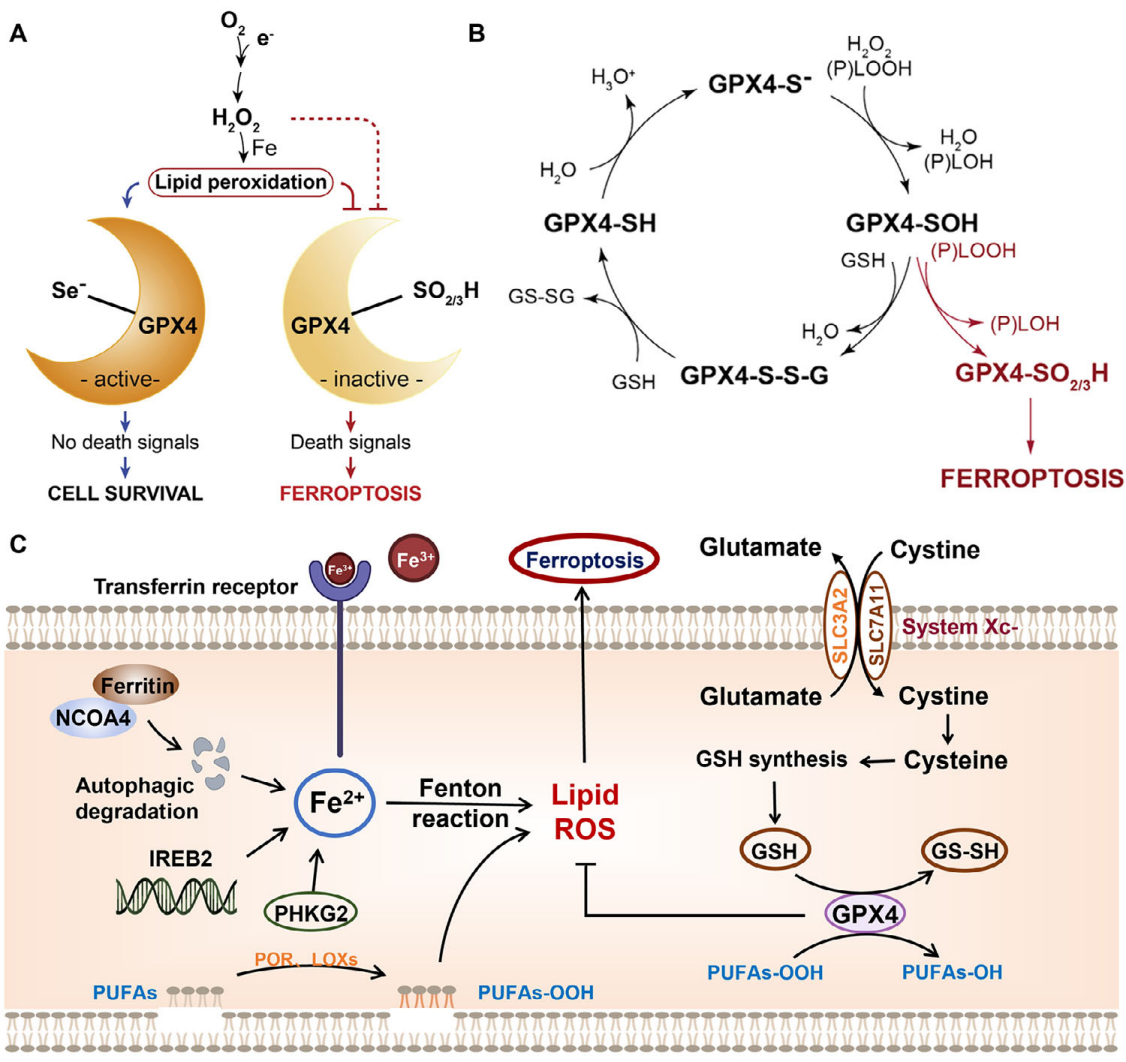
The neuroprotective activity mechanism of Se by inhibiting ferroptosis in stroke. (A,B) GPX4 prevents oxidative stress‐induced ferroptosis [78]. Copyright 2018, Cell Press. (C) Pathological cascade induced by ferroptosis [79].

**FIGURE 3:**
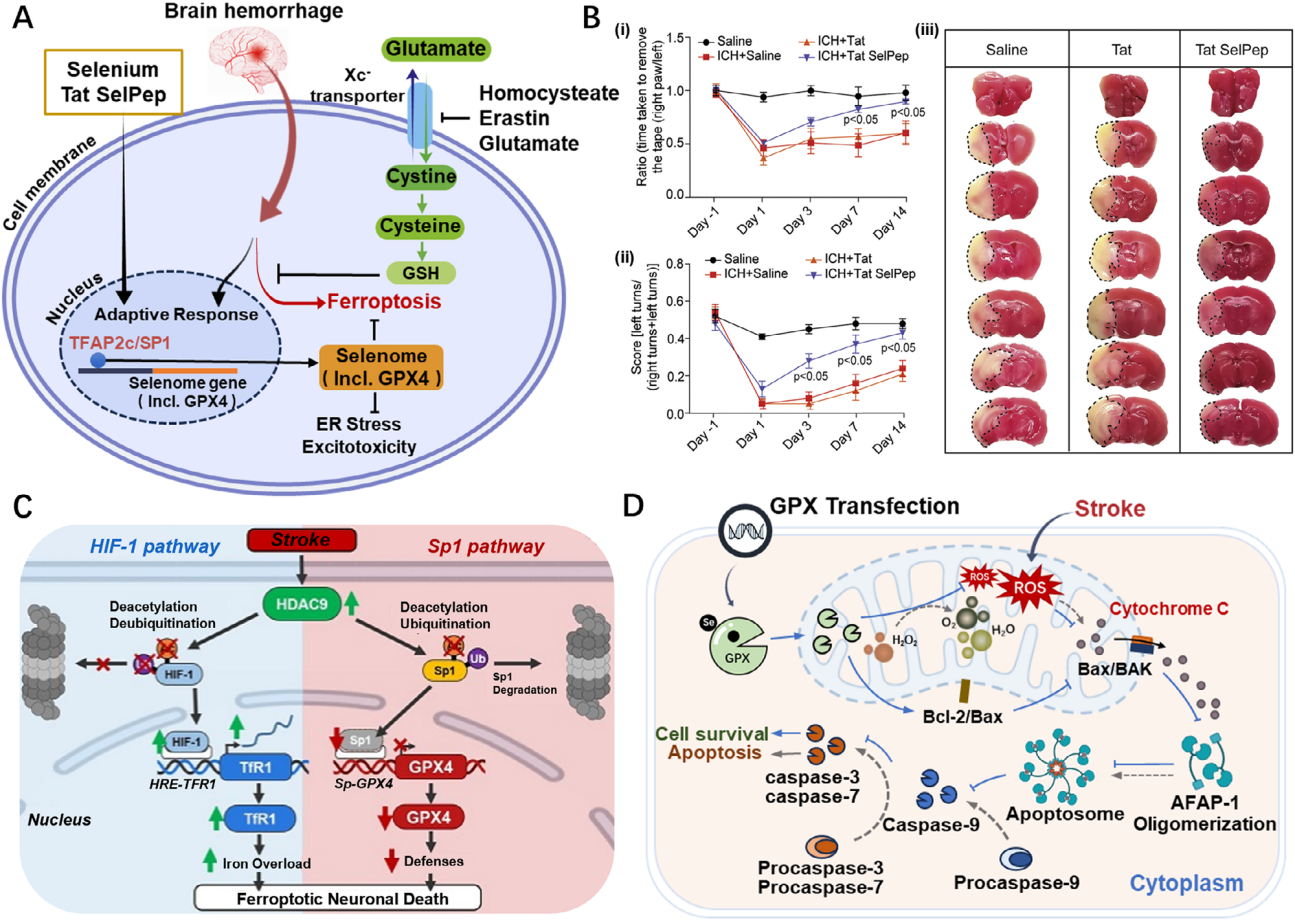
The application of selenoprotein activity and treatment by Se in stroke. (A) Pharmacological Se supplementation effectively inhibits ferroptosis in hemorrhagic stroke; (B) Intraperitoneal injection of Tat SelPep can improve sensory (i) and spatial (ii) neglect and reduce the size of cerebral infarction (iii) in mice [85]. Copyright 2019, Cell Press. (C) HDAC9 knockdown alleviates neuronal ferroptosis by enhancing GPX4 [90]. (D) GPX overexpression improves neuronal survival after stroke by clearing ROS and inhibiting cytochrome C release [91]. Copyright 2003, American Heart Association.

Previous studies have shown that ferroptosis is the secondary cause of cell death in neurons after stroke and is characterized by the excessive accumulation of lipid peroxides and ROS [80]. Interestingly, Xiao et al. demonstrated that BSA‐stabilized Se nanoparticles (BSA‐SeNPs) can inhibit secondary brain damage caused by ferroptosis after hemorrhagic stroke by activating the Nrf2/GPX4 pathway [81]. Se can protect particular neurons depressing induced by ferroptosis through adding selenocysteine during the translation of the selenoprotein, GPX4 (Figure 2) [78]. This adaptive response is regulated by factors, for example, cellular tumor antigen p53 and nuclear factor erythroid 2‐related factor 2, and is critical in preventing ferroptosis to promote cell survival [82, 83, 84]. However, Se deficiency enhances this response, requiring additional Se to enhance selenoprotein transcriptional regulation. Recently, Alim et al. demonstrated that ferroptosis stimulation can cause the nervous system to produce selenoproteins (including GPX4) during transcription in vitro and in vivo (Figure 3A) [85]. In addition, sodium selenite supplementation can inhibit ferroptosis and non‐ferroptosis‐dependent cell death by coordinating the activation of transcription factors such as transcription factor family activator protein 2 and specific protein 1 (Sp1) to up‐regulate the transcriptional expression of selenoproteins. Polypeptides containing selenocysteine can improve brain function recovery by delivering Se to the ventricles after stroke to improve, the adaptive transcriptional response linked to ferroptosis. Particularly, in a hemorrhagic stroke model, the intraperitoneal injection of a single dose of Se upregulated the expression of Sp1 and GPX4, promoting the recovery of neurological function and reducing the size of cerebral infarction (Figure 3B). Furthermore, weighted correlation network analysis revealed an up‐regulation in the expression of genes linked to resistance to endoplasmic reticulum stress and neuroexcitatory toxicity after Se supplementation. Notably, selenoproteins can shield neurons against excitatory neurotoxicity, ER stress, and ferroptosis. Overall, this study highlights the potential of Se supplements as a neuroprotective agent for stroke treatment, with important guiding implications in nutritional care and the subsequent treatment of cerebral hemorrhage.

Histone deacetylases (HDACs) can regulate transcription processes by catalyzing the removal of acetyl groups from the lysine residues of histone and non‐histone proteins [86]. HDAC9 is overexpressed in neurons during stroke and exerts neurotoxic effects by inhibiting autophagy, activating inhibitor kappa B alpha /nuclear factor kappa‐B (IκBα/NF‐ĸb) and mitogen‐activated protein kinases (MAPKs) signaling pathways, and reducing the expression of miR‐20a, upregulating its target gene Neurod1 [87, 88, 89]. According to Sanguigno et al., HDAC9 interacts with hypoxia‐inducing factor 1 (HIF‐1) and Sp1, transcriptional activators of the GPX4 and transferrin receptor 1 (TfR1) genes, respectively, in nerve cells exposed to oxygen‐glucose deprivation/reperfusion (Figure 3C) [90]. This binding results in elevated levels of HIF‐1, which promote the transcription of the ferrophilic TfR1 gene and decreased levels of the Sp1 protein. The reduced Sp1 levels also lead to the downregulation of the iron‐resistant GPX4 gene. Oppositely, neuronal death induced by ferroptosis stimulation was reduced by silencing HDAC9, HIF‐1, TfR1, Sp1, and GPX4. Overall, stroke‐induced neuronal ferroptosis was suppressed by inhibiting the downregulation of GPX4.

Researchers further investigated the function of GPX in protecting neurons following stroke. Hoehn et al. investigated the protective effects of gene therapy, namely the overexpression of the GPX in stroke induction, which effectively decreased the release of the proapoptotic protein cytochrome C and increased the neuronal survival ratio (Figure 3D) [91]. Even after transfection following ischemia, GPX could prevent stroke with a 9–11 h therapeutic window. GPX overexpression dramatically decreased the cytoplasmic translocation of cytochrome C and raised the percentage of B‐cell lymphoma‐2 positive cells compared to the control vector‐transfected cells. Furthermore, GPX overexpression inhibited processes associated with apoptosis by downregulating Bcl2‐Associated X and triggering caspase‐3. In summary, this study revealed that the overexpression of GPX can be utilized in neuroprotection to reverse ischemia injury in stroke patients.

### 2.2 Selenium Therapeutic Agent in Alzheimer's Disease

**FIGURE 4:**
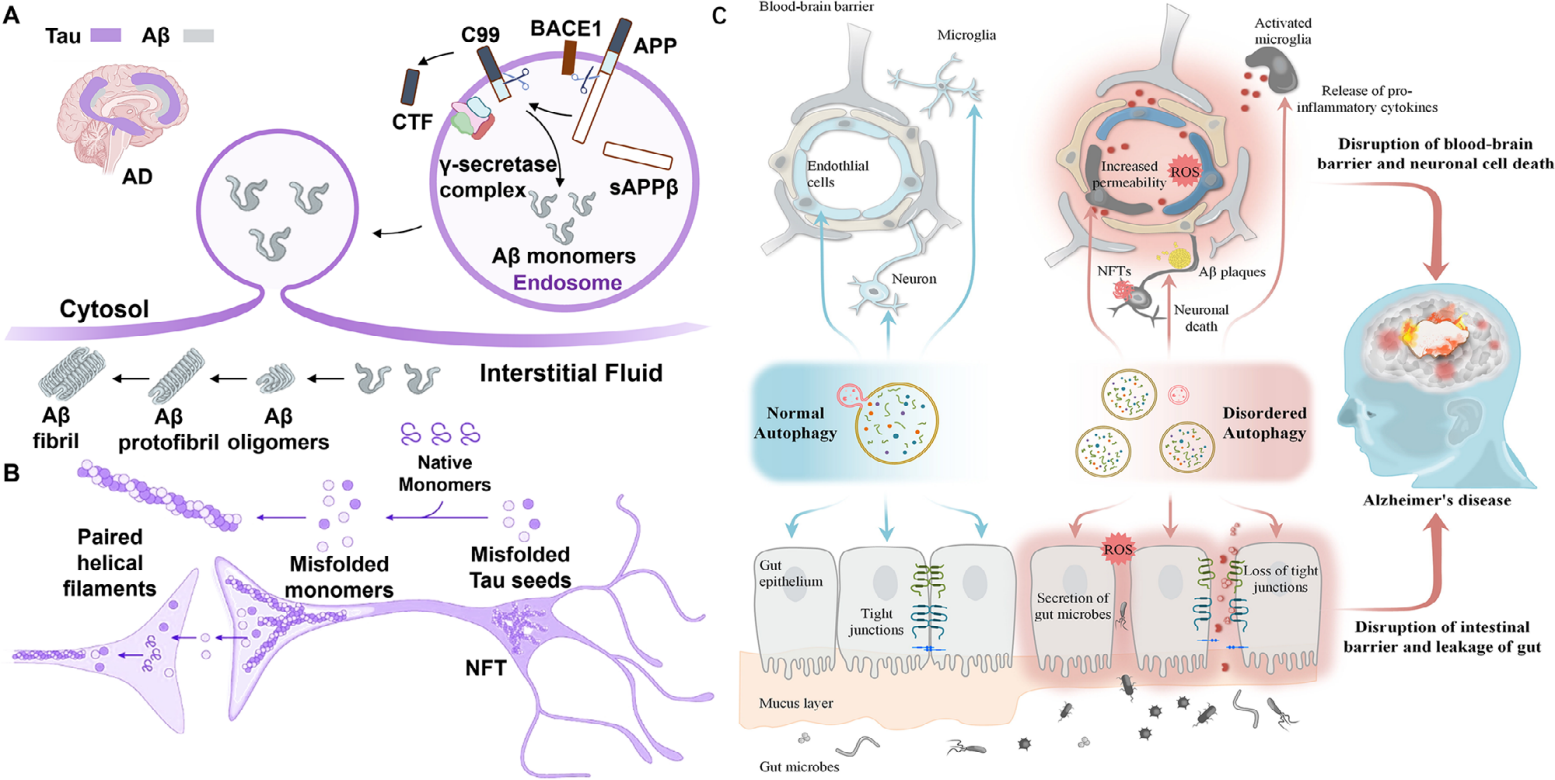
The main pathological mechanism of AD: (A,B) Deposition of Aβ protein and Tau protein aggregation [97]. Copyright 2019, Cell Press. (C) Brain‐gut axis autophagy dysfunction [101, 102]. Copyright 2020, Wiley‐VCH.

Alzheimer's disease (AD) is a neurodegenerative disorder, clinically characterized by progressive memory loss and cognitive decline [92]. There are several theories regarding the pathogenesis of AD, with the most prevailing being the amyloid β‐protein (Aβ) cascade hypothesis. The Aβ hypothesis posits an imbalance between the production and clearance of Aβ that leads to neuronal degeneration and dementia [63, 93, 94]. Another popular theory is the tubulin‐associated unit (Tau) protein hypothesis, which suggests that the hyperphosphorylated Tau protein affects the stability of neuronal cytoskeleton tubulin. This would result in the formation of neurofibrillary tangles (NFTs) and the subsequent disruption of normal neuronal and synaptic functions [95]. Clinical pathological results have revealed the presence of senile plaques formed by Aβ deposits in the brain tissue of AD patients. It was also found that Tau proteins are composed of hyperphosphorylated plaques and that NFTs are formed from Tau cells (Figure 4A,B) [96, 97]. Current strategies aimed at clearing or reducing the abnormal accumulation of Aβ or Tau proteins in the brain to treat AD, though their efficacy remains limited [98, 99]. AD is caused by oxidative stress and its products that damage DNA, lipids, proteins, and many macromolecules, aggravating the development of neurodegenerative diseases. During AD disease, oxidative stress acts within the cell bilayer and serves as a source of ROS, causing lipid peroxidation to occur. The Aβ peptide induces the formation of various oxidative adducts that may disrupt synaptic transmission and result in cell apoptosis. Furthermore, oxidative stress can exacerbate the cytotoxicity of Aβ. This is similar to the relationship between α‐Syn protein and oxidative stress [100]. In recent years, the neuroprotective activity of selenoproteins has led to increased interest in Se for the treatment of AD.

Studies revealed that the concentration of Se in the brain tissue of AD patients was only 60% that of compared with age‐matched healthy individuals [103]. Both β‐secretase and γ‐secretase are crucial in Aβ production, cleaving amyloid precursor protein (APP), which also generates the soluble peptide APPβ (sAPPβ), to generate Aβ1‐40 and Aβ1‐42. Sodium selenite can confer neuroprotection against lipid peroxidation and Aβ toxicity by inhibiting endogenous γ‐secretase activity [104]. Song et al. reported that Se administration in AD model mice led to the significant down‐regulation of β‐secretase levels and a consequent reduction in cerebral Aβ production [105]. Furthermore, SelP can regulate microtubule assembly by interacting with the C‐terminal domain of α‐tubulin and mitigate the intracellular ROS burden by interacting with Tau proteins, Ca^2+^, and polyamines. Thus, SelP can preserve microtubule structure and function in the AD model [106]. These findings suggest that Se may modulate the pathogenesis of AD by inhibiting Aβ aggregation and Tau protein phosphorylation, potentially altering the disease course.

**FIGURE 5:**
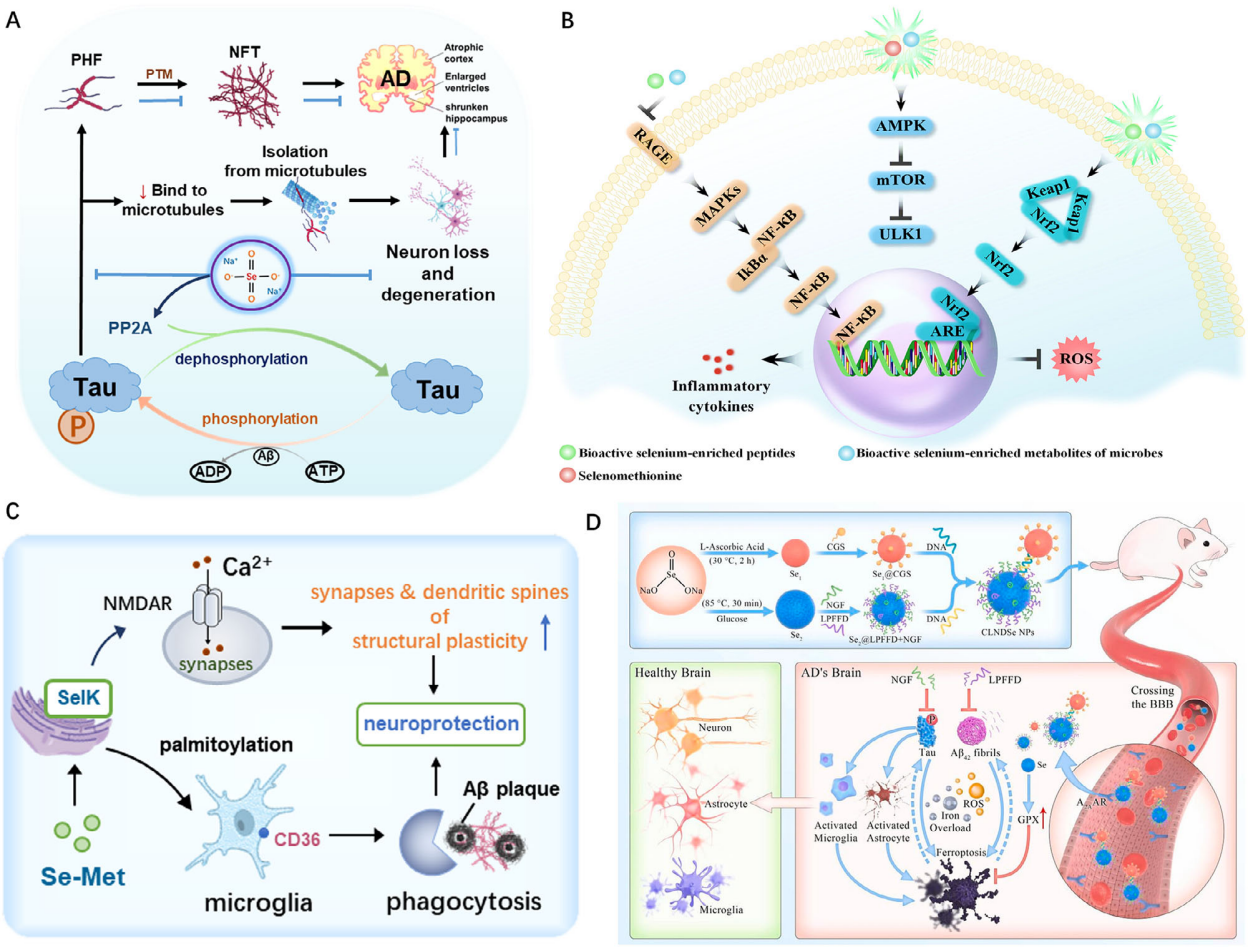
Application of Se in the treatment of AD. (A) Selenate treatment prevented axonal degeneration of different neuronal populations in K3 mice [107]. Copyright 2010, National Academy of Sciences. (B) Autophagy, antioxidant, and anti‐inflammatory signaling pathways regulated by Se‐enriched bioactive ingredients targeted AD [114]. (C) The overexpression of SelK in microglia of AD mice suggests that SelK is involved in the regulation of immune function in the central nervous system [117]. Copyright 2019, Mary Ann Liebert. (D) Multi‐functional double Se nanospheres (CLNDSe) can regulate the expression of GPX4 to treat AD by disrupting the aggregation of Aβ42 and inhibiting hyperphosphorylation of Tau protein [118]. Copyright 2023, Elsevier.

Previous research on AD therapy has primarily targeted Aβ, while relatively few therapeutic strategies have been developed to address Tau pathology. Through compound screening, Eersel et al. identified sodium selenate as a promising agent that can mitigate neurocognitive impairment, reversing memory, and motor deficits in mice. This effect was mediated by the activation of protein phosphatase 2A (PP2A) and the subsequent dephosphorylation of specific Tau epitopes in SHSY‐5Y cells expressing the human Tau subtype (Figure 5A) [107]. Notably, selenate can significantly reduce the number of Tau inclusions among the subhippocampal and midbrain regions in K3 mice. This finding suggests that the supplementation of Se effectively reduces Tau phosphorylation and deposition, alleviates the formation of pathological spheroids, and prevents axonal degeneration in diverse neuronal populations in vivo. The double staining of human Tau (HT7) and Ser422‐phosphorylated Tau (pS422) showed a marked decrease in the number of pS422‐positive neurons, a pathological marker of severe Tau, and Gallyas‐positive NFTs after treating Tau transgenic mice with selenate. These results underscore a reduction in Tau phosphorylation within the brain, particularly in CA1 neurons, highlighting the potential of selenate as a therapeutic agent in modulating Tau pathology in Alzheimer's disease.

In addition to Tau and Aβ, autophagy is closely associated with the pathogenesis of AD (Figure 4C). Researchers have demonstrated that autophagy dysfunction, driven by ROS accumulation and neuroinflammation, accelerates the progression of AD [108]. Consequently, regulating autophagy levels presents a promising strategy for mitigating AD [109]. As research into the nutritional role of Se has advanced, the significant biological activities of Se‐enriched foods and their active components (Se‐enriched ingredients, SEIs, such as seleno‐amino acids and selenoproteins) have become evident. These activities include the regulation of autophagy, oxidation–reduction equilibrium, and anti‐inflammatory signaling pathways [110, 111, 112, 113]. Ding et al. reviewed and analyzed the impact of active SEIs on neuronal and intestinal stress responses by Se‐enriched food, revealing that SEIs not only exert anti‐inflammatory and antioxidant effects by inhibiting Nrf2 activated by the MAPKs/NF‐κB pathway, but also enhance Aβ clearance and alleviate memory impairment by activating the AMP‐activated protein kinase/ mammalian target of the rapamycin (AMPK/mTOR) pathway (Figure 5B) [114]. Song et al. also found that selenomethionine (Se‐Met) therapy can improve cognitive deficits in triple transgenic AD (3xTg‐AD) mice through two patterns: (1) the inhibition Tau hyperphosphorylation by regulating Akt (protein kinase B), glycogen synthase kinase 3β and PP2A activities; (2) autophagy initiation can activate the AMPK‐mTOR signaling pathway, and promote Tau clearance in AD neurons [115, 116]. These results have highlighted the potential of Se‐containing amino acids to intervene in autophagy dysfunction and oxidative stress, revealing a novel mechanism of Se‐promoted cognitive function in AD patients.

Overactivation of the NMDAR system induces glutamate neurotoxicity, which leads to neuronal death and synaptic loss in AD treatment. Therefore, achieving a balanced regulation of synaptic and extra‐synaptic NMDARs presented potential therapeutic value for AD [119, 120]. Song et al. found that Se‐Met modulates NMDAR2‐dependent calcium influx in 3 × Tg‐AD mice by upregulating the expression of SelK, a selenoprotein localized in the endoplasmic reticulum and highly expressed in immune cells, involved the proliferation and differentiation of various immune cells [43, 121, 122]. This intervention improves the structural plasticity of synapses and dendritic spines, enhancing their cognitive function [117]. Notably, SelK was significantly downregulated in the brains of AD patients and mice models [123, 124]. As macrophages of the central nervous system, microglia play a crucial role in regulating neuroimmune response and cognitive function [125]. Nevertheless, toxic Aβ fibrils not only cause the Aβ phagocytosis disorder of microglia but also stimulate neuronal Aβ secretion [126]. The overexpression of SelK affects central nervous system immune function by modulating the migration and phagocytosis of microglia [44]. Zhang et al. confirmed that SelK deficiency impaired their microglial phagocytosis of Aβ and exacerbated the cognitive deficits of 5xFAD (Transgenic mice with five familial AD) mice, while overexpression of SelK can reverse this process by incubating with selenomethionine (Figure 5C) [127]. Mechanistic studies revealed that SelK participated in the palmitoylation of CD36 through DHHC6, regulating the localization of CD36 to the plasma membrane of microglial cells. This modulation enhanced Aβ phagocytosis by microglia and contributed to the attenuation of AD progression. These findings have elucidated the regulatory role of SelK in the interaction between microglia and Aβ aggregates, highlighting its impact on neuroimmune homeostasis and providing new insights into the role of selenoprotein in combating AD.

Signaling indicative of ferroptosis, including the inactivation of GPX4, were observed in the brains of AD patients [128, 129, 130]. Moreover, Aβ42 facilitates the reduction of Fe^3+^ to Fe^2+^, promoting ferroptosis and causing neuronal damage [131]. Wang et al. reported multifunctional double selenide nanospheres can achieve the dual effect of destroying Aβ aggregation and inhibiting Tau hyperphosphorylation (Figure 5D) [118, 132]. Se nanoparticles (SeNPs) modified with the LPFFD peptide, which specifically binds to the central sequence of Aβ42, selectively interacted with Aβ42 to promote the disruption of Aβ42 aggregates and inhibit ferroptosis [133, 134, 135]. Additionally, SeNPs modified with nerve growth factor, an inhibitor (NGF), an inhibition of Tau hyperphosphorylation, significantly reduced Tau phosphorylation and glial cell activation in APP/PS1 mice. According to the study, these multifunctional double selenide nanosphere NPs effectively restored GPX1/4 expression levels, enhanced the antioxidant capacity of brain tissue, diminished neuronal damage and neurofibrillary tangles, prevented neuronal ferroptosis, and ultimately ameliorated cognitive deficits in APP/PS1 mice. Thus, Se shows potential therapeutic benefits in modulating AD pathology by inhibiting Aβ and Tau abnormalities and enhancing neuroprotection, while, selenoproteins can affect microglial activity and inhibit ferroptosis to improve cognitive function.

### 2.3 Mechanisms and Therapeutic Potential of Selenium in Parkinson's Disease

The primary pathological change in Parkinson's disease (PD) is the aggregation of α‐synuclein fibers to form, forming Lewy bodies. This leads to the degeneration and necrosis of dopaminergic neurons in the midbrain substantia nigra, which significantly reduces dopamine levels in the striatum [136]. Additionally, oxidative stress plays a significant role in the development of Parkinson's disease. Oxidative stress is the most important of these, occurring when ROS overwhelm cellular antioxidant capacity. The accumulation of cytotoxic compounds leads to the breakdown of proteins, the failure of enzymes, and the degradation of lipids [137]. There is evidence that mitochondrial dysfunction plays an important role in the pathophysiology of sporadic and monogenic Parkinson's disease. Further, complex I deficiency is also involved in the pathogenesis of Parkinson's disease, in addition to mitochondrial DNA mutations [138]. According to literature research, dopamine replacement therapy is the most commonly used clinical treatment, providing symptomatic relief without altering disease progression. Beyond pharmacological interventions, researchers are investigating new therapies for PD, including immunotherapy and stem cell therapy [139, 140]. However, immunotherapy has not shown substantial therapeutic efficacy due to its high cost and off‐target effects, while stem cell therapy has failed in clinical trials and not only failed to achieve the intended beneficiary effects but also faces ethical controversies and challenges related to immune rejection [141]. Thus, researchers have focused on the pathogenic mechanisms associated with oxidative stress and potential therapeutic strategies for its inhibition.

**FIGURE 6:**
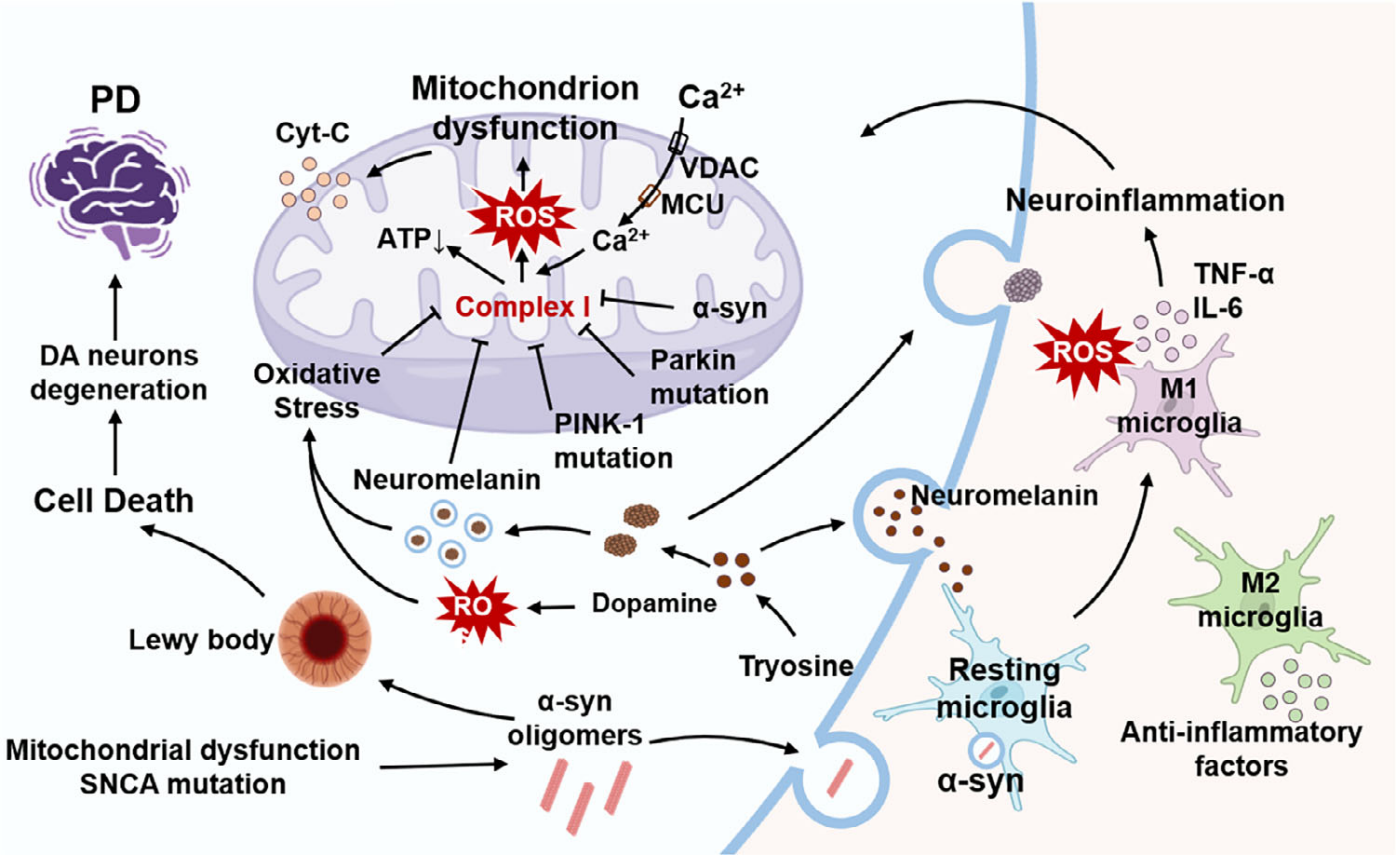
Multifaceted pathological mechanisms of PD. (1) Mechanism of mitochondrial dysfunction, including α‐synuclein, gene mutations, and oxidative stress suppress complex I [142]. (2) Abnormal dopamine metabolism‐induced oxidative stress and neuromelanin accumulation [143, 144]. (3) Microglia was activated by neuromelanin, α‐synuclein, and dead neurons [145]. The interplay of these mechanisms led to the degeneration of dopaminergic neurons, collectively driving the onset and progression of PD.

The pathogenesis of PD is complex, affected by the interaction of oxidative stress, mitochondrial dysfunction, neuroinflammation, abnormal dopamine metabolism, and other factors (Figure 6) [143, 144, 146, 147, 148, 149, 150]. Researchers found that the imbalance of trace elements, such as Se, iron, and copper, in the body may play an important role in the occurrence and development of PD including oxidative stress and damaging the nervous system [151]. In the substantia nigra of PD patients, complex I activity and glutathione were significantly reduced, while highly active quinone molecules are spontaneously produced [152]. Therefore, the imbalance of antioxidant defense systems may be the core factor in the onset and progression of PD [153].

Recent studies have provided deeper insights into the role of Se in PD. Transcriptomic analysis of Se in PD mouse models revealed unexpected differences in the expression of selenoproteins, as almost all selenoproteins were down‐regulated in the substantia nigra. Imam and Ali observed that Se deficiency caused severe chemical damage to the dopaminergic nerve endings of PD mice [154]. While, Ellwanger et al. found that selenite could reduce the degree of DNA damage and alleviate the symptoms of bradykinesia in vivo [155]. Sun conducted a survey in 48 states of the United States to study the relationship between the concentrations of Se, strontium, and magnesium in soil and the mortality rate of PD in the United States. This result has shown that relatively high concentrations of Se and magnesium in soil can benefit PD patients [156]. Additionally, GPX1^−/−^ mice induced with 1‐methyl‐4‐phenyl‐1,2,3,6‐tetrahydropyridine were prone to dopamine depletion and increased 3‐nitrotyrosine production in the striatum [157]. Therefore, selenoproteins capable of regulating REDOX homeostasis may be key mediators in this neurodegenerative disease.

**FIGURE 7:**
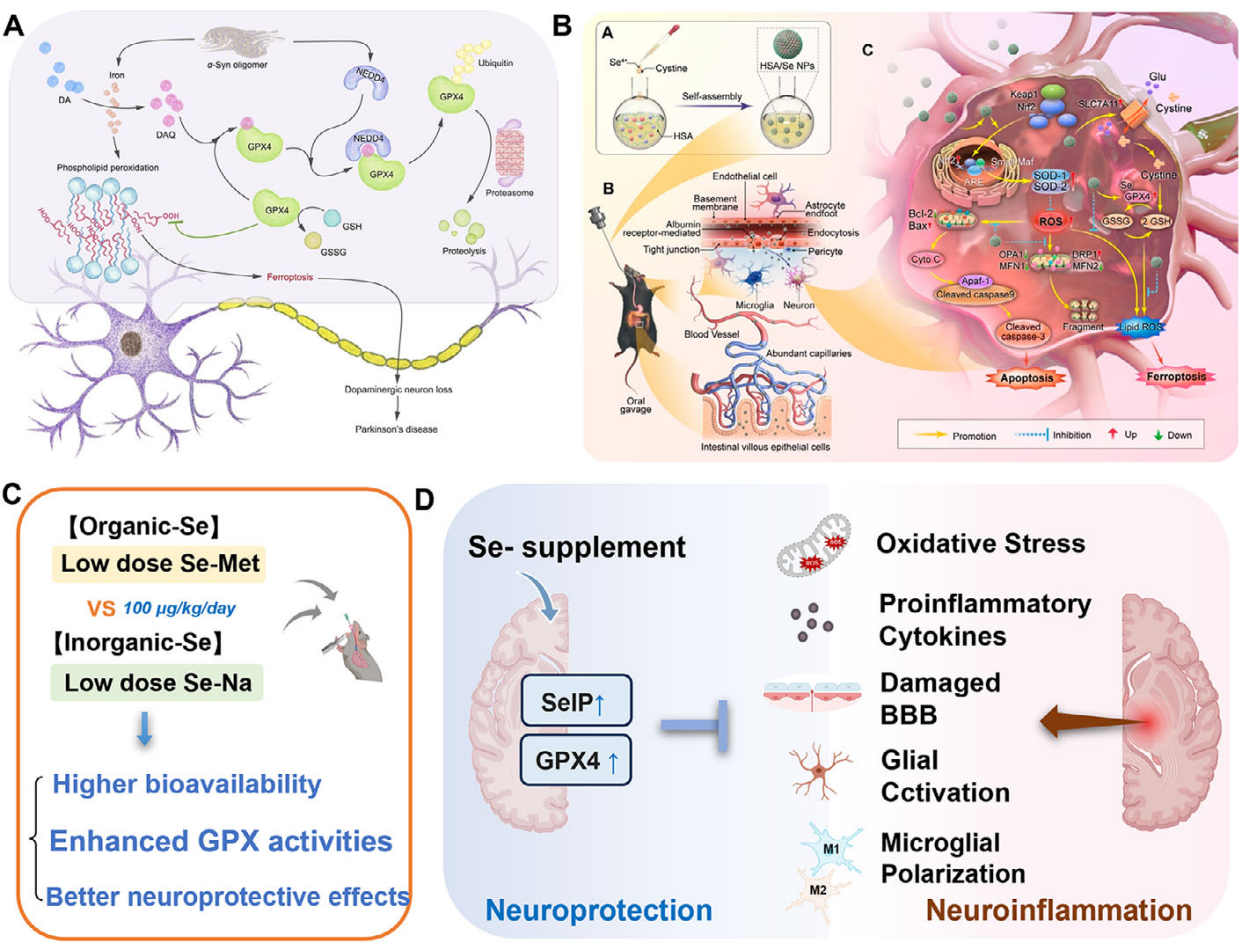
Therapeutic application of Se in Parkinson's disease. (A) Pathogenesis of PD through dopaminergic neuron degeneration, which is exacerbated by the loss of antioxidant enzyme GPX4 activity [159]. (B) Human serum albumin/Se nanoparticles provided neuroprotection to dopaminergic neurons via modulated Keap1‐Nrf2‐SOD signaling pathways [160]. Copyright 2023, American Chemical Society. (C) Administration of low‐dose Se–Na significantly improved motor performance [161]. (D) Se supplementation alleviated neuroinflammation by enhancing SelP and GPX4 expression in the hippocampus of mice [32]. Copyright 2023, Elsevier.

Notably, the pathogenesis of PD has been linked to ferroptosis and lipid peroxidation (Figure 7A) [158]. α‐Synuclein plays a critical role by inducing the expression of genes associated with ferroptosis through the upregulation of RSL3. This process leads to the accumulation of 4‐hydroxynonenal (4‐HNE) and malonaldehyde (MDA), an increase in TFR protein levels, and the reduced expression of GPX4, ultimately resulting in ferroptosis. GPX4, a pivotal regulator, is essential for protecting brain tissue from ferroptosis. Additionally, α‐synuclein can induce an elevation in neuronal precursor cells expressed developmentally downregulated 4 (NEDD4) levels [159]. In the iron‐rich environment of the substantia nigra, unstable iron accelerates the spontaneous oxidation of DA to form electron‐deficient dopamine quinone (DAQ). DAQ then interacts with GPX4 by marking it for degradation by ubiquitin proteins through a process facilitated by NEDD4. The dysfunction of GPX4 contributes to lipid peroxidation to proceed unchecked, thereby contributing to ferroptosis.

Other studies have revealed the relationship between GPX4 and PD. GPX4 was co‐localized with melanin in the substantia nigra, elevating GPX4 expression levels was elevated in the axons and cortex of the malnourished patients [20]. In oxidative stress response, GPX4 expression was increased with the separation of Parkinson's disease protein 7 (PARK7) from its mRNA [162]. Finally, oxidized dopamine targeted mitochondrial GPX4 and reduced its activity in dopaminergic neurons [163]. Therefore, maintaining GPX4 activity is a promising strategy for early intervention in PD.

Even though existing therapeutic strategies can alleviate PD symptoms, they cannot prevent dopamine neuron loss. The study by Xu et al. looked at a custom‐made human serum albumin (HSA)‐based selenium nanosystem (HSA/Se nanoparticles, HSA/Se NPs) that could get through the intestinal epithelial barrier and the blood‐brain barrier to treat Parkinson's disease. Specifically, HSA/Se NPs inhibit mitochondrial‐related apoptosis through the activation of the Keap1‐Nrf2‐SOD pathway and the Bcl‐2‐cytochrome C‐caspase pathway, suppressing ferroptosis by activating the GPX4‐SLC7A11‐GSH pathway in a PD model. As a result, HSA/Se NPs were found to be less toxic and more effective than other selenium species, as well as having the ability to bypass the BBB and intestinal epithelial barrier in order to enrich dopamine neurons, which inhibited neurotoxicity and ameliorated both behavioral deficits and dopamine neuronal death in PD model mice. (Figure 7B) [160].

It is worth noting that the form and dose of medication determine the biological role of Se in vivo. Sun et al. compared the neuroprotective effects of high dose (1000 µg Se/kg/day) and low dose (100 µg Se/kg/day) of sodium selenite and organic selenomethionine in PD models and found that sodium selenite had a better neuroprotective effect (Figure 7C) [25]. Specifically, low doses of sodium selenite were more effective than Se‐Met in preventing dopaminergic cell death and mitigating the decline of dopamine and its metabolites in vivo to increase GPX activity. However, it should be noted that Se–Na is significantly more toxic than Se‐Met, which might explain why the low‐dose results were effective but not the high‐dose results. Additionally, this report has demonstrated the Se–Na higher bioavailability of Se–Na compared to Se‐Met, elucidating its more pronounced neuroprotective effects.

Furthermore, oral Se intake at appropriate doses has been shown to have beneficial effects on the treatment and progression of neurological diseases in both human and animal models [164]. Specifically, Se's neuroprotective function is mainly achieved by optimizing the function of antioxidant selenoproteins that protect the neuronal cytoskeleton through a reasonable intake of Se. Liang et al. reported the absorption of dietary supplementation with functionalized selenite nanoparticles by the gastrointestinal epithelium through activating transport and passive diffusion pathways (Figure 7D) [32]. SelP, a major Se transporter, subsequently binds to the surface receptor APOE2 through clathrin‐dependent endocytosis to transport Se to the brain, thereby inducing an overall increase in the expression of selenoproteins in the hippocampus of mice, especially SelP and GPX4. Selenoproteins play a neuroprotective role in the brain injury of neuroinflammatory mice by down‐regulating the expression of caspase8, NF‐ĸB and inflammatory cytokines (IL‐1β, TNF‐α) as well as reducing lipopolysaccharide (LPS)‐induced ROS production in Se supplementation (Se‐sup) mice, increasing the total antioxidant capacity of cells. In addition, Se supplementation improved the blood–brain barrier (BBB) integrity and tolerance to neuroinflammation by counteracting the downregulation of occludin and zonula occludens 1 (ZO‐1) expression induced by LPS and reducing albumin infiltration. Notably, Se supplementation enhanced neurological function by increasing the expression of postsynaptic density protein‐95 (PSD‐95) and brain‐derived neurotrophic factor (BDNF). Overall, Se played a neuroprotective role primarily by reducing oxidative stress and alleviating neuroinflammation in PD.

### 2.4 Exploring the Role of Selenium in Huntington's Disease

Huntington's disease (HD) is an autosomal dominant neurodegenerative disease caused by a genetic mutation of abnormal Huntington proteins (HTT) that accumulate in brain tissue and cause neuronal dysfunction [165, 166]. HTT is widely expressed in all organs of the body, including the central nervous system, affecting the development of the nervous system, cell entosis and secretion, and the inhibition of apoptosis. Abnormal Htt protein in HD patients have many repeated trinucleotide (CAG) repeats, which are prone to the adhesion, aggregation, and eventual death of nerve cells [167]. As there is no specific treatment for this disease, and symptomatic treatment options are often adopted, such as dopamine receptor blockers and cholinergic drugs or the supplementation of central gamma‐aminobutyric acid [168]. Most clinical trials on the treatment of HD focused on HTT, such as HD single nucleotide polymorphism (HD‐SNP) targeting, however, the therapeutic effect is lacking [169].

Emerging research suggests the benefits of selenoproteins in mitigating the oxidative stress observed in HD. Through proteomic analysis of the striatum and cortex in the brain tissue of HD patients, Sorolla et al. observed increased levels of antioxidant defense proteins, such as GPX1, GPX2, and GPX6 in the striatum and cortex [170]. Additionally, the activity of other antioxidant enzymes such as mitochondrial superoxide dismutase and catalase increased in HD. These findings indicate oxidative stress as a crucial pathological mechanism in the progression of HD. Consequently, enhancing the oxidative stress resistant ability of HD patients could improve mitochondrial dysfunction and facilitate nervous system repair. Researchers have demonstrated that Se supplementation can decrease the aggregation of mutant huntingtin and reduce levels of oxidized glutathione in the brains of HD mice [171]. Additionally, Se supplementation has been shown to alleviate oxidative stress and lipid peroxidation, effectively mitigating quinoline‐induced neurodegeneration in HD model mice.

**FIGURE 8:**
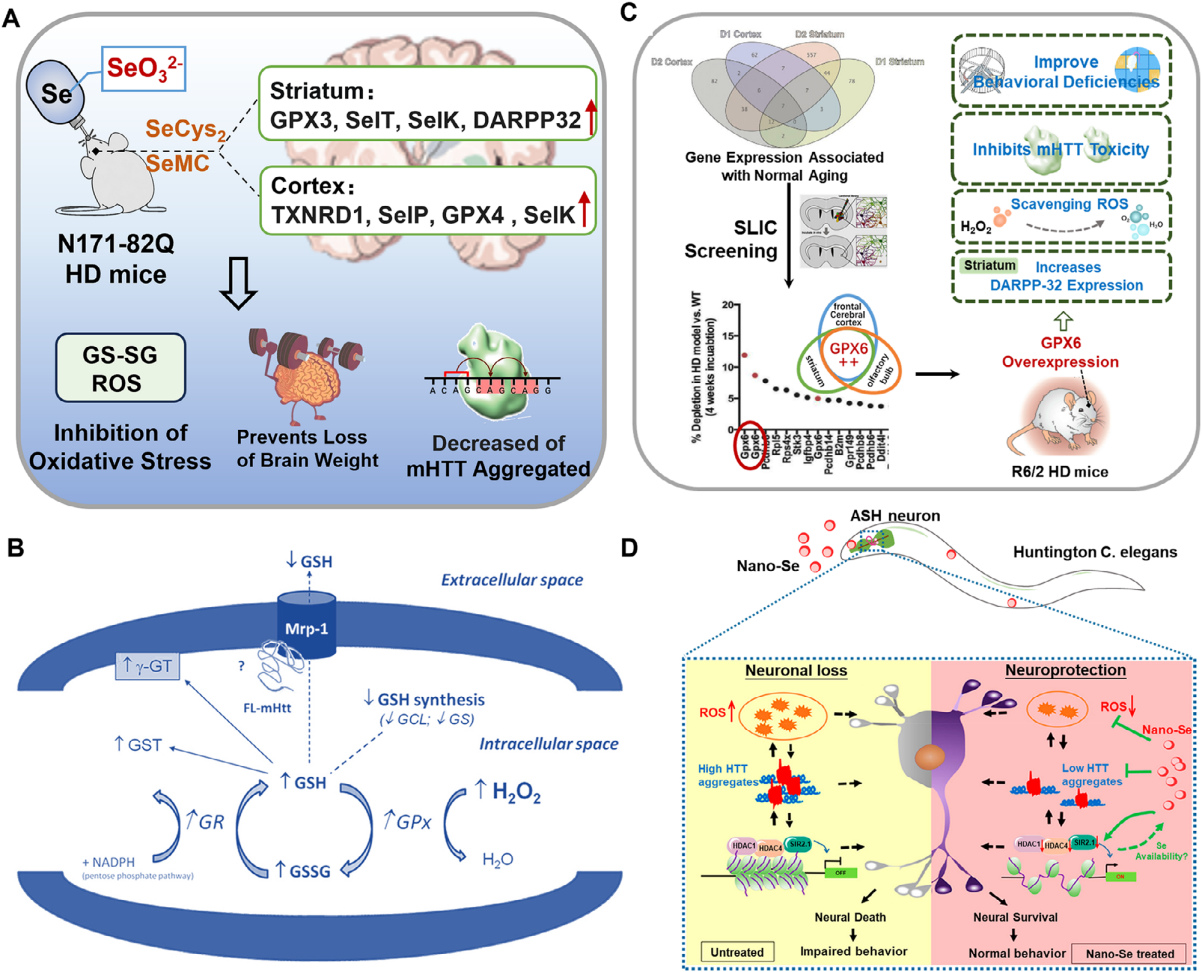
Application of Se in the treatment of Huntington's disease. (A) Sodium selenite alleviated oxidative stress, neurodegeneration and mutant Huntington protein aggregation in HD mice by regulating selenoproteins [172]. Copyright 2014, Elsevier. (B) GSH‐related antioxidants are increased in the striatum and cortex of FL‐mHtt mice [173]. Copyright 2012, Elsevier. (C) GPX6 as a suppressor of mutant Huntingtin toxicity [174]. Copyright 2014, National Academy of Sciences. (D) Nano‐Se alleviated oxidative stress and neurological dysfunction in transgenic HD models of *Caenorhabditis elegans* [168]. Copyright 2019, American Chemical Society.

Se supplementation can effectively reverse the decline in selenoproteins expression in HD, which can regulate homeostasis of REDOX [175]. Lu et al. found that the aggregate burden of mutant HTT decreased after supplying 1.0 ppm sodium selenite in water. It was also observed decreased oxidized glutathione (GSSG), a marker of oxidative stress, and increased DARPP32 expression in the striatum, a marker of HD transcription dysregulation (Figure 8A) [172]. By analyzing the effects of sodium selenite treatment on the transcriptional levels of selenoproteins coding in the brain, researchers have reported increased TrxR1 and SelP levels in the cortex, activating GPX3 and SelT in the striatum, as well as reduced levels of GPX4 in the cortex and SelK. Furthermore, sodium selenite prevented 92 % of the average weight loss that occurred in the brains of HD mice between 6 and 14 weeks. Sodium selenite not only increased the GPX3 transcription levels in the striatum of HD mice, but also reversed HD‐related changes in liver Se levels and the plasma glutathione of N171‐82Q mice (a common mouse model of HD), affecting the expression of brain selenoproteins.

The activation and enhancement of selenoproteins promoted the expression of other antioxidant‐related proteins. Sorolla et al. observed increased mRNA levels of GPX1, catalase and Cu‐SOD /Zn‐SOD in the striatal cells of FL‐mHtt [170]. Ribeiro et al. investigated the endogenous antioxidant system expressing full‐length mutant Htt (FL‐mHtt) and found that the activity of the glutathione redox cycle was enhanced in vitro, with increased levels ROS, caspase‐3 activity intracellular GSH and GSSG levels (Figure 8B) [173]. Further assessment of GPX and glutathione reductase (GRed) activities revealed that, along with changes in GSH and GSSG, the total GPX activity and mitochondrial GPX activity were significantly elevated in mutant cells compared to the control group. The changes in GRed activity were consistent with those of GPX. Specifically, GPX catalyzed the conversion of GSH and H_2_O_2_ into GSSG and H_2_O, while GRed used GSSG and nicotinamide adenine dinucleotide phosphate (NADPH) to generate GSH and NADP. These findings suggest that striatal cells expressing FL‐mHtt exhibited a heightened antioxidant defense profile, primarily through the upregulation of the glutathione redox cycle, including the antioxidant selenoproteins GPX and GRed. Although this enhancement in antioxidant defenses was insufficient to fully counteract oxidative stress and apoptosis in HD striatal cells, it partially prevented extensive neuronal death and significant mHtt aggregation, reducing the initial ROS production in mutant cells.

Shema et al. demonstrated that GPX6 genetically interacts with mutant Huntingtin. It was found that ROS, particularly hydrogen peroxide, heightens the toxicity of mutant Huntingtin, which is a phenomenon that worsens with age (Figure 8C) [174]. Additionally, the reduction of the GPX6 gene exacerbated the toxicity of the mutant Huntingtin protein. Notably, GPX6 in the olfactory bulb, striatum, and frontal cerebral cortex increased with age. All GPX6‐targeted shRNAs exhibited synthetic lethality with mutant Huntingtin, which was similarly or less affected in the R6/2 model (a common mouse model of HD), suggesting specific interactions with GPX6. Furthermore, the overexpression of GPX6 elevated dopamine‐ and cAMP‐regulated phosphoprotein, Mr 32 kDa (DARPP‐32) expression in the R6/2 model, a molecular marker of HD progression. Therefore, the overexpressing GPX6 provided protective effects against both behavioral and molecular phenotypes linked to HD progression.

Insufficient Se levels in the brains of HD patients suggested that maintaining Se homeostasis in the brain may be beneficial in mitigating the occurrence and development of HD. However, different types of Se compounds exert different physiological effects owing to different metabolic pathways [176, 177, 178]. Among these, nano‐Se demonstrates superior antioxidant capacity and biosafety compared to other forms of Se. Consequently, Cong et al. evaluated the therapeutic potential of nano‐Se using a transgenic HD model in *Caenorhabditis elegans* (Figure 8D) [168]. Research also showed that low doses (2 µM) of nano‐Se significantly alleviated neuronal death and behavioral dysfunction in transgenic worms by repairing oxidative stress damage. Mechanistic investigations revealed that nano‐Se inhibits Huntington protein aggregation, reducing neuronal oxidative stress‐related mRNA levels, and downregulating the expression of histone deacetylase family members (HDAC1, HDAC4, and silent information regulator sirtuin 1). These results highlight the significant therapeutic potential of nano‐Se in treating HD and offer valuable insights for the rational design of nano‐Se nanoparticles and other Se compounds to improve HD treatment.

### 2.5 Selenium and Other Central Nervous System Disease

**FIGURE 9:**
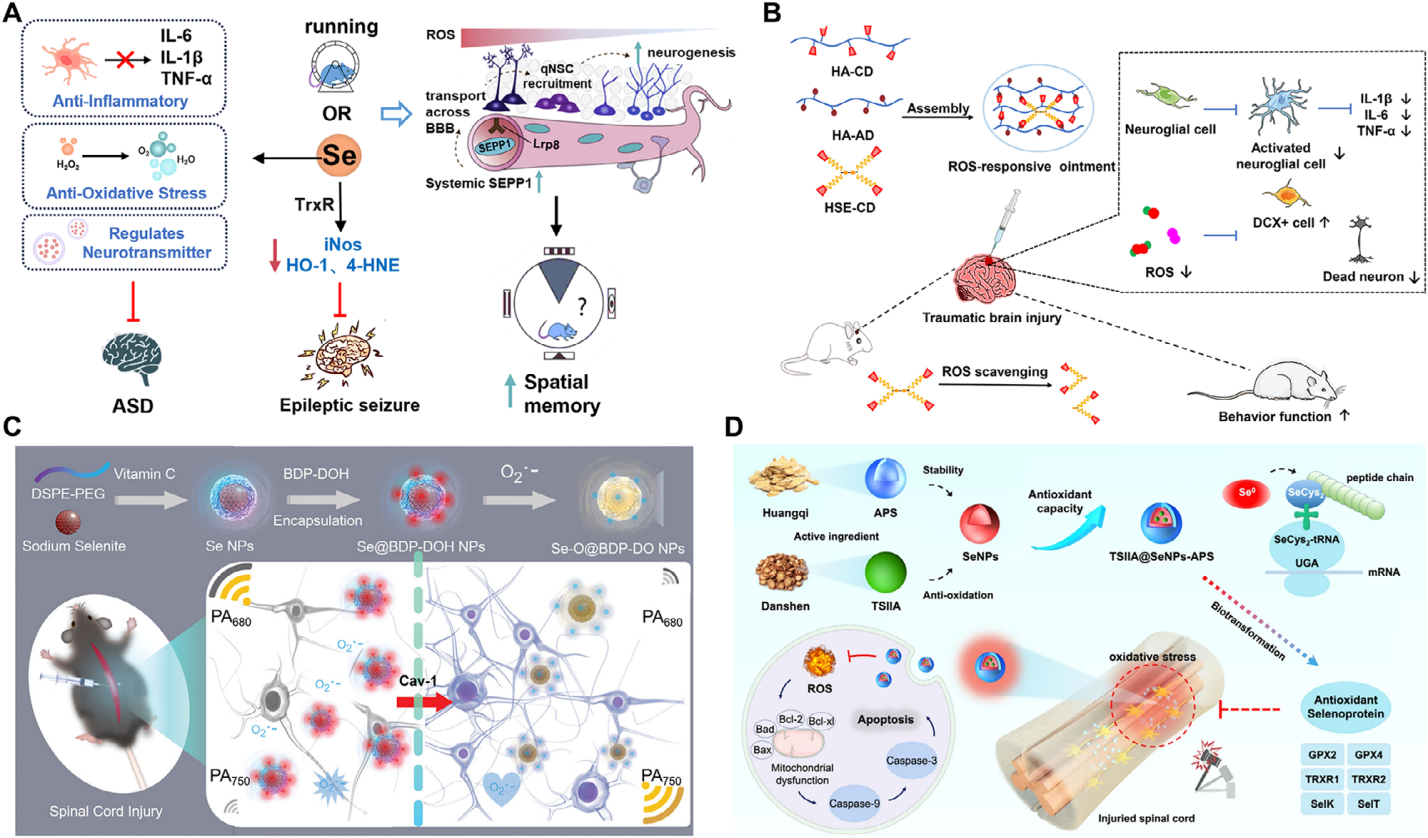
Application of Se in the treatment of other central neuronal diseases. (A) Mechanisms of Se in the treatment of ASD and Epileptic seizure by anti‐inflammatory, anti‐oxidative stress and regulates neurotransmitter [15, 179, 180]. (B) Selenium‐containing ROS‐responsive ointment suppresses oxidative stress and inflammation to provide synergistic treatment of TBI [185]. Copyright 2024, Elsevier. (C) Se@BDP‐DOH using hydrophobic–hydrophobic interactions to accurately map the redox status and eliminate oxidative stress in SCI [187]. Copyright 2023 Wiley‐VCH. (D) TSIIA@SeNPs‐APS can increase GPX activity and decrease MDA content, possibly through its metabolism to L‐Selenocystine (SeCys_2_) and by regulating antioxidant selenoproteins to protect spinal cord neurons from oxidative stress‐induced damage [188]. Copyright 2020, BioMed Central.

In addition to the diseases mentioned previously, Se is closely associated with the onset and progression of various neurological disorders (Figure 9A). For example, Waiker et al. utilized an SelP knockout model to show that SelP and its receptor, the low‐density lipoprotein receptor‐associated protein 8 (LRP8), were essential for exercise‐induced increases in adult hippocampal neurogenesis [179]. Dietary supplementation with Se and mimicking the effects of exercise, can restore neurogenesis and counteract cognitive decline related to aging and hippocampal damage. Furthermore, Se supplementation administered through endothelin‐1 (ET‐1) and inducible nitric oxide synthase (iNOS) mediated hippocampal injury significantly alleviated cognitive and memory impairments caused by the injury. In patients with severe epilepsy, increased expression of antioxidant selenoproteins, such as SelW, GPX1, and TrxR1, in surgically removed brain tissue suggested enhanced Se utilization in epilepsy. Additionally, Se supplementation has been shown to reduce seizures, with recurrence observed upon discontinuation of the supplement. In individuals with major depressive disorder (MDD), serum Se levels were notably lower compared to healthy controls. Higher levels of trace elements such as iodine, copper, Se, and molybdenum may help prevent MDD progression [180]. Compared with normal children, the autism spectrum disorder (ASD) had lower Se concentration in the hair and blood [181, 182]. After supplying Se, social function, repetitive behavior, and cognitive function were significantly improved in vivo [15].

Traumatic brain injury (TBI) describes serious damage to brain tissue caused by an external force or jolt, which may result in short‐ or long‐term neurological deficits, including secondary damage that occurs as a result of the primary injury [183]. The pathological cascade processes such as oxidative stress and inflammatory responses are considered to be important causes of secondary damage to brain tissue. Oral supplementation of *N*‐acetylcysteine (NAC) and Se after TBI can alleviate oxidative stress by inhibiting ROS production and increasing the expression level of GPX, and at the same time downregulate the level of pro‐inflammatory (IL‐1β) cytokines and upregulate the level of anti‐inflammatory (IL‐4) cytokines to alleviate inflammatory stimulation, thereby showing a neuroprotective effect on TBI [184]. Hu et al. also reported that ROS‐responsive ointment (R‐O) was effective in treating TBI. R‐O can reduce cell apoptosis at the injury site by reducing ROS levels, but also alleviate the inflammatory response by increasing the ratio of M2/M1 microglia and inhibiting the expression of pro‐inflammatory factors, thereby promoting nerve regeneration and functional recovery (Figure 9B) [185]. As part of the central nervous system, the spinal cord is closely connected to the brain. The spinal cord injury (SCI) is a sudden deterioration of the central nervous system caused by oxidative stress and inflammation. In order to effectively treat SCI in the emergency setting, it is critical to prevent secondary injuries caused by ROS and to monitor and assess the recovery after SCI [186]. Herein, in order to effectively inhibit the damage caused by oxidative stress in SCI, an emergency treatment strategy is developed based on the Se‐based antioxidant system. Ji et al. developed an emergency treatment and photoacoustic severity assessment (ETPSA) strategy using Se@BDP‐DOH nanoparticles. This strategy allows for real‐time evaluation of SCI severity through photoacoustic imaging while modulating caveolin 1 (Cav1) levels to alleviate oxidative stress. Meanwhile, Se@BDP‐DOH nanoparticles have been shown to promote neuronal survival, motor function recovery, and spinal cord tissue regeneration in the SCI mouse model, offering a promising therapeutic avenue for patients with SCI (Figure 9C) [187]. In addition, Se and traditional Chinese medicine composite nanomaterials have also been effective in SCI treatment. Rao et al. have demonstrated that Se nanoparticles (TSIIA@SeNPs‐APS), derived from the active ingredients of traditional Chinese medicine, not only alleviate mitochondrial damage and S‐phase cell cycle arrest in PC12 cells by reducing ROS levels at the cellular level but also enhance the expression of the antioxidant selenoprotein GPX and decrease lipid peroxidation products (MDA) at the animal level, thereby exhibiting therapeutic effects on SCI (Figure 9D) [188]. This indicates that Se was found to play a protective role in regulating neurotransmitter levels, reducing oxidative stress, alleviating neuroinflammation, and rescuing neuronal damage, thus providing a basis for targeted treatment of ASD, TBI, SCI, and Epileptic seizure.

## 3 Conclusion and Perspective

This review provides a comprehensive overview of Se in its underlying mechanism and neuroprotective mechanisms. Additionally, it highlights the role of selenoproteins, the direct mediators of Se's functions, in the pathology of nervous system disorders and explores their active mechanisms (Table 2). In particular, different Se‐based drugs can be metabolized in the body, resulting in H_2_Se and selenophosphate, which are then converted into amino acids containing Se, such as selenocysteine, which are then converted into selenoproteins. By investigating the mechanisms and applications of Se and selenoproteins in central nervous system diseases, to inspire innovative designs for Se‐based therapeutics, and facilitate their clinical translation. Despite the promising potential of Se and selenoproteins in neurological diseases, further research and exploration are required to fully utilize their therapeutic capabilities.

**TABLE 2:** Summarization of selenium‐related application for central nervous system diseases.

Dimension	Influence factors	Results	Model	Reference
Ischemic stroke	GPX1 knockout	Inhibiting neuronal damage by GPX1	MCAO model in vivo	[73]
Ischemic stroke	GPX1 knockout	GPX1 regulates ROS‐related inflammation	I/R model in vivo	[77]
Oxidative stress	Selenocysteine	Protect neurons by depressing ferroptosis and regulating GPX4	Oxidative stress by H_2_O_2_ and GPX4 knockout mice	[78]
Hemorrhagic stroke	BSA‐SeNPs	Inhibiting ferroptosis by activating the Nrf2/GPX4 pathway	Hippocampus‐intracerebral hemorrhage model in vivo	[81]
Stroke	Sodium selenite	Driving transcription of GPX4 via a Sp1‐mediated pathway	MCAO model in vivo	[85]
Ischemic stroke	siHDAC9	Inhibiting the downregulation of GPX4 can suppress stroke‐induced neuronal ferroptosis.	MCAO model in vivo	[90]
Ischemic stroke	GPX overexpression	Decreasing the release of cytochrome C and increasing the neuronal survival ratio	MCAO model in vivo	[91]
Alzheimer's disease	Sodium selenate	Reducing tau hyperphosphorylation and abrogates NFT formation	K3 mice in vivo	[107]
Alzheimer's disease	SEIs	Antioxidant, anti‐inflammation, and autophagy modulating effects	AD mice in vivo	[114]
Alzheimer's disease	Se‐Met	Upregulating SelK levels and restoring synaptic deficits	3 × Tg‐AD mice	[117]
Alzheimer's disease	CLNDSe NPs	Inhibiting Aβ_42_ aggregation and attenuated Aβ_42_‐induced neural toxicity	APP/PS1 mice	[118]
Alzheimer's disease	SelK knockout	Enhanced ER stress and increased apoptosis in neurons	SelK knockout mice	[123]
Alzheimer's disease	SelK knockout or overexpression	SelK enhances microglial Aβ phagocytosis	SelK knockout and SelK overexpression mice	[127]
Alzheimer's disease	SeNPs	Inhibits Aβ aggregation and crosses the BBB	PC12 cell in vitro	[133]
Parkinson's disease	Sodium selenite	Maintenances locomotor activity and the integrity of leukocytes DNA	PD model in vivo	[155]
Parkinson's disease	HSA/Se NPs	Overcome the intestinal epithelial barrier and blood‐brain barrier	MPTP model mice	[160]
Parkinson's disease	Se–Na and Se‐Met	Se–Na had higher bioavailability than Se‐Met	PD model in vivo	[25]
Parkinson's disease	Sodium selenite	Antioxidant, anti‐inflammation, and upregulated SelP and GPX4 levels	LPS‐induced Se‐sup mice neuroinflammation model in vivo	[32]
Huntington's disease	Proteomic analysis	GPX1 and GPX6 activity increased	HD patients	[170]
Huntington's disease	Sodium selenite	Decreasing mutant huntingtin aggregate burden and brain oxidizing glutathione levels	N171‐82Q model in vivo	[172]
Huntington's disease	Glutathione redox cycle	FL‐mHtt increases levels of oxidative stress	HD knock‐in mice	[173]
Huntington's disease	GPX6 overexpression	Alleviating both behavioral and molecular phenotypes	HD model in vivo	[174]
Traumatic brain injury	*N*‐Acetylcysteine or Se	Alleviating oxidative stress and inflammatory	Marmarou's weight drop model in vivo	[184]
Traumatic brain injury	R‐O ointment	Reducing ROS and inflammatory for promoting nerve regeneration	Marmarou's weight drop model in vivo	[185]
Spinal cord injury	Se@BDP‐DOH	Modulating caveolin 1 and promoting neuronal survival	Spinal cord contusion modeling in vivo	[187]
Spinal cord injury	TSIIA@SeNPs‐APS	Activating GPX and decreasing MDA	Spinal cord contusion modeling in vivo	[188]

Future studies are needed to address the following aspects. First, most studies consider that Se‐based drugs exert neuroprotective effects through their conversion into selenoproteins, which regulate oxidative stress and facilitate Se transport. A deeper investigation into the intricate mechanisms of selenoprotein activation is crucial to better understand the onset and progression of neurological disorders. Uncovering novel pathogenic mechanisms related to these diseases would allow for the development of Se‐based drugs that can inhibit the progression of neurologic damage. It is necessary to design drugs that target disorders of the central nervous system based on the pathogenic mechanisms of these diseases and the specific characteristics of the blood‐brain barrier. For example, we can increase the enrichment of Se‐containing drugs in the central nervous system by changing the size of SeNPs modifying targeted molecules of the blood‐brain barrier, or using Se‐containing drugs to reduce the aggregation of Aβ protein based on AD pathogenic mechanism. Such therapies can be designed to activate selenoproteins expression, mitigate Se‐related toxicity, and achieve neural specificity. With targeted design and thorough research, these Se‐based drugs hold considerable promise for enhancing neuroprotection in neurological disease treatment. Although, numerous Se‐based drugs have been synthesized, a more comprehensive understanding of their safety profiles, metabolism, and physiological functions in vivo is essential. Finally, the cost‐effective production of Se‐based drugs must be optimized for future development. Ultimately, rigorous validation and optimization are necessary to ensure the safety, efficacy, and affordability of Se‐based therapies. By addressing these critical challenges, the translation of Se‐based drugs into practical neuroprotective treatments for neurological diseases can be achieved.

## Conflicts of Interest

The authors declare no conflicts of interest.

## Data Availability

The data that support the findings of this study are available from the corresponding author upon reasonable request.
